# Cellular and molecular features of COVID-19 associated ARDS: therapeutic relevance

**DOI:** 10.1186/s12950-023-00333-2

**Published:** 2023-03-20

**Authors:** Gaetano Scaramuzzo, Francesco Nucera, Alessio Asmundo, Roberto Messina, Matilde Mari, Federica Montanaro, Matt D. Johansen, Francesco Monaco, Guido Fadda, Giovanni Tuccari, Nicole G. Hansbro, Philip M. Hansbro, Trevor T. Hansel, Ian M. Adcock, Antonio David, Paul Kirkham, Gaetano Caramori, Carlo Alberto Volta, Savino Spadaro

**Affiliations:** 1 Department of Translational Medicine, University of Ferrara, Ferrara, Italy; 2 Department of Emergency, Section of Intensive Care and Anesthesia, Azienda Ospedaliera-Universitaria Sant’Anna, Ferrara, Italy; 3 Pneumologia, Dipartimento di Scienze Biomediche, Odontoiatriche e delle Immagini Morfologiche e Funzionali (BIOMORF), Università di Messina, Messina, Italy; 4 Medicina Legale, Dipartimento di Scienze Biomediche, Odontoiatriche e delle Immagini Morfologiche e Funzionali (BIOMORF), Università di Messina, Messina, Italy; 5 Intensive Care Unit, Dipartimento di Patologia Umana e dell’Età Evolutiva Gaetano Barresi, Università di Messina, Messina, Italy; 6 Centre for Inflammation, School of Life Sciences, Faculty of Science, Centenary Institute and University of Technology Sydney, Sydney, NSW Australia; 7 Chirurgia Toracica, Dipartimento di Scienze Biomediche, Odontoiatriche e delle Immagini Morfologiche e Funzionali (BIOMORF), Università di Messina, Messina, Italy; 8 Section of Pathological Anatomy, Department of Human Pathology of Adult and Developmental Age “Gaetano Barresi”, University of Messina, Messina, Italy; 9 Medical Research Council and Asthma, UK Centre in Allergic Mechanisms of Asthma, London, UK; 10 Airway Disease Section, National Heart and Lung Institute, Imperial College London, London, UK; 11 Department of Biomedical Sciences, Faculty of Sciences and Engineering, University of Wolverhampton, West Midlands, Wolverhampton, UK

**Keywords:** COVID-19, SARS-CoV-2, ARDS, Biomarkers, Cytokines, Chemokines

## Abstract

The severe acute respiratory syndrome-coronavirus-2 (SARS-CoV-2) infection can be asymptomatic or cause a disease (COVID-19) characterized by different levels of severity. The main cause of severe COVID-19 and death is represented by acute (or acute on chronic) respiratory failure and acute respiratory distress syndrome (ARDS), often requiring hospital admission and ventilator support.

The molecular pathogenesis of COVID-19-related ARDS (by now termed c-ARDS) is still poorly understood. In this review we will discuss the genetic susceptibility to COVID-19, the pathogenesis and the local and systemic biomarkers correlated with c-ARDS and the therapeutic options that target the cell signalling pathways of c-ARDS.

## Introduction

The coronavirus disease-19 (COVID-19) pandemic remains a major challenge for healthcare systems worldwide. Severe acute respiratory syndrome-coronavirus-2 (SARS-CoV-2) infection can be asymptomatic or cause a disease (called COVID-19) of different grades of severity. The main cause of severe COVID-19 and death is represented by pneumonia with acute (or acute on chronic) respiratory failure until the acute respiratory distress syndrome (ARDS) which requires often intensive care unit (ICU) admission and mechanical ventilation [1].

The molecular pathogenesis of COVID-19-related ARDS (by now termed c-ARDS) is still poorly understood. We will review here the literature on the genetic susceptibility, the pathogenesis and the local and systemic biomarkers correlated with c-ARDS compared with ARDS non-COVID-19-related including potential new therapeutic options targeting the cell signalling pathways of c-ARDS.

### Genetic susceptibility in developing of c-ARDS

Many single nucleotide polymorphisms (SNPs) and several autosomal or X-linked deficiency involving both innate and adaptative immune system and their intracellular pathways, may have a role in the development of c-ARDS [2, 3] and are summarized in Table 1. Specifically, the genetic background can increase the cellular entry and the replication of SARS-CoV-2 or the immunological response to the infection.

**Table 1:** Predisposing or protective genetic factors in the development of severe COVID-19

Genetic factors	Predisposing	Protective
ACE2 SNPs	rs2285666 (3’ fold)	
TMPRSS2 SNPs		rs12329760 (OR: 0.89)
HLA system haplotype and alleles	Class HLA I: A*11:01 (OR:2.23); B*51:01 (OR: 3.38); C*04:01 (2’ fold); C*14:02 (OR 4.72) Class HLA II: rs9271609 HLA-DRB1 (OR 1.1)	HLA I: A*02:01; A*03:01 (only in homozygosis); A*68:02 (OR 0.448); B*14 (OR: 0.434)
TLR3 mutation and SNPs	Autosomal dominant mutations present only in severe patients (p.Ser339fs/WT, p.Pro554Ser/WT, p.Trp769*/WT, p.Met870Val/WT); rs3775291 (r = 0.33)	
TLR7 mutation and SNPs	Recessive X-linked mutations present only in severe patients (L134P/Y, N158Tfs11*/Y, L227fs*/Y, D244Y/Y, F310L/Y, L372M, I505T/Y, H630Y/Y, I657T/Y, F670Lfs*8, F670Lfs*8, K684*/Y, P715S/Y, H781L/Y, L988S/Y, L988S/Y and M854I;L988S/Y)	
IFNAR2 SNPs	rs2236757 (OR 1.2), rs13050728 (OR 1.3)	
OAS-1, -3 SNPs	rs10735079 (OR 1.3)	
IRF7	Autosomal recessive mutations (p.Pro364fs/p.Pro364f;p.Met371Val/p.Asp117Asn) Autosomal dominant mutations (p.Arg7fs/WT, p.Gln185*/WT, p.Pro246fs/WT, p.Arg369Gln/WT, p.Phe95Ser/WT)	
IFNs	Serum auto-antibodies against IFN-I (IFNα,IFNω) and IFN-II; rs28368148 of IFNA10 (OR 1.7)	Absence of serum auto-antibodies against IFNs I (IFNα, IFNω) and IFN II
IFITIM3	rs12252 (GA and GG genotype OR 2.2; C genotype OR 1.6)	
IL-10RB	rs8178521 (OR 1.2)	
CCR5	rs9845542 (OR 1.33), rs12639314 (OR 1.23) and rs35951367 (OR 1.32)	deletion of CCR5Δ32 rs333 (OR:0.66)
ABO blood group and SNPs related	A group; rs657152 (OR 1.7), rs 9,411,378 (OR 1.2); FUT2 rs 48,697,960 (CT genotype OR 1.1)	O group

Data obtained from: [4, 5, 7–10, 12, 13, 16, 18, 22, 24, 28, 30, 33]. Abbreviation: *ACE *Angiotensin I-converting enzyme, *FUT2 *Fucosyltransferase 2, *HLA *Major histocompatibility complex, *IFN *Interferon, *SNP *Single nucleotide polymorphism, *IFNAR2 *Interferon-alpha, -beta, and -omega receptor 2, *IL10RB *Interleukin-10-receptor-beta, *IRF7 *IFN regulatory factor 7, *OAS *Oligoadenylate synthase, *OR *Odds ratio, *r *Correlation, *TLR *Toll-like receptor, *TMPRSS2* Transmembrane serine protease 2

### SNPs and deficiency of genes that increase SARS-CoV-2 entry and replication into airway cells

#### ACE2 gene SNPs

Several angiotensin I-converting enzyme (ACE)2 SNPs (located in Xp22.2) have shown an increased receptor affinity for the SARS-CoV-2 proteins, therefore facilitating its access into cells. For example, the ACE2 SNP rs2285666 GG genotype (prevalence of 0.2% and 0.55% of European and Asian populations, respectively) can triple the susceptibility to the development of severe COVID-19, whereas the TT genotype increases the risk of hospitalization and acute respiratory failure [4, 5].

### TLRs SNPs and deficiency

Autosomal dominant deficiencies of the toll-like receptor (TLR)3 expression (located in 4q35.1) including p.Ser339fs/WT, p.Pro554Ser/WT, p.Trp769*/WT, p.Met870Val/WT are found in ~ 3.5% of severe COVID-19 patients (and also in life-threatening influenza-associated pneumonia) but absent in asymptomatic/mild SARS-CoV-2 infected patients [6]. In addition, SNP rs3775291 of TLR3 is weakly correlated with both susceptibility of SARS-CoV-2 infection and mortality [7]. Moreover, also very rare X-linked TLR7 variants (located in Xp22.2) in ~ 1% male patients such as L134P/Y, N158Tfs11*/Y, L227fs*/Y, D244Y/Y, F310L/Y, L372M, I505T/Y, H630Y/Y, I657T/Y, F670Lfs*8, F670Lfs*8, K684*/Y, P715S/Y, H781L/Y, L988S/Y, L988S/Y and M854I; L988S/Y were not found in asymptomatic/mild SARS-CoV-2 infected patients, but only in some severe/critical COVID-19 patients [8].

### Pro-inflammatory mediators signalling abnormalities

Some patients with severe COVID-19, ~ 12.5% and ~ 2.6% in males and females respectively, have serum IgG neutralizing autoantibodies anti-interferon (IFN)α and/or IFNω not detectable in the asymptomatic/mild SARS-CoV-2 infection [9].

The SNP rs2236757 characterized by A allele of the interferons (alpha, -beta, and -omega) receptor 2 (IFNAR2), located in 21q22.11, is weakly associated with its decreased expression and increased risk of severe COVID-19 [10, 11]. Other SNPs of IFNAR2 such as rs13050728 expressing T allele and rs33242905 are correlated to weak increased risk of severe COVID-19 [7, 12, 13].

Moreover, an analysis of the Arabic population has shown that the rs12252 SNP within the interferon-induced transmembrane protein (IFITM)3 gene (genotype GA or GG) is strongly associated with increased risk of hospitalization due to COVID-19 and mortality, correlating with decreased serum IFNγ levels [14]; whereas 2 studies on Caucasian and Chinese population have shown that this SNP was strongly correlated to increased risk of hospitalization when characterized by the C allele, in particular when in homozygosis [15, 16]. However, another study evaluating a German population has not confirmed these data showing that this SNP is not associated with increased risk of severe COVID-19 [17].

Another study has shown that the rs10735079 SNP within the antiviral oligoadenylate synthase (OAS)-1 and-3 genes that are IFN-induced, are weakly correlated with increased risk of hospitalization during SARS-CoV-2 infection [18, 19].

In addition, the rs28368148 (CG genotype) correlating with the interferon-alpha-10 (IFNA10) gene was found strongly correlating with severe COVID-19, whereas the rs8178521 (CT genotype) of the IL-10-receptor-beta (IL-10RB) gene was found weakly correlated with severe COVID-19 [13].

Moreover, although there is not a direct correlation with the SARS-CoV-2 infection, both the rs6734238 (located on 2q14, GA genotype) of the IL-1R gene and the rs4537545 (located on 1q21, TC genotype) of the IL-6R gene are correlated with increased serum levels of IL-6 that are correlated with COVID-19 severity and in addition represent a main target in the COVID-19 therapy (see below) [20].

Both autosomal recessive deficiencies of IFN regulatory factor 7 (IRF7), located in 11p15.5, including p.Pro364fs/p.Pro364f and p.Met371Val/p.Asp117Asn and autosomal dominant IRF7 including p.Arg7fs/WT, p.Gln185*/WT, p.Pro246fs/WT, p.Arg369Gln/WT, p.Phe95Ser/WT deficiencies are found in ~ 3.5% of severe COVID-19 patients (and also in life-threatening influenza-associated pneumonia) but absent in asymptomatic/mild SARS-CoV-2 infected patients [6].

Moreover, an analysis of Italian population has shown that 3 SNPs of the chemokine CC-motif receptor (CCR)5 gene such as rs9845542, rs12639314, and rs35951367 (this when characterized by genotype GT) are weakly associated to increased risk of development of severe COVID-19 correlating with decreased lung expression of CCR5 [21].

### HLA system SNPs and alleles

Several SNPs and alleles of both human leukocyte antigen (HLA) class I and II (respectively located in 6p22.1 and 6p21.32) increase the risk of severe COVID-19, although it is still now not fully clear the mechanism induced severe COVID-19 HLA-mediated, growing evidence suggest that this can be induced by different SARS-CoV-2 antigen presentation HLA-mediated that correlate with abnormal immune cells response [12, 22–24].

Class I HLA alleles such as HLA-A*11:01, HLA-B*51:01, HLA-B*54, HLA-C*14:02, were found to be strongly correlated with increased risk of hospitalization due to SARS-CoV-2 infection [24–27].

In addition, the class I HLA-C*04:01 allele is correlated with increased risk of development of COVID-19 with double risk of onset acute respiratory failure needed intubation [28].

Moreover, patients with a decreased class II HLA-DR cluster 1 showed increased risk of developing severe COVID-19 [22, 28], and in particular rs9271609 (TC genotype) of the HLA-DRB1 was weakly correlated with severe COVID-19 [13].

Similarly, although there is not a direct correlation with the SARS-CoV-2 infection, the rs660895 SNP (located on 6p21, genotype GA) of the HLA-DRB1 and HLA-DRB5 are correlated with increased serum levels of IL-6 [20].

### ABO blood gene

The ABO blood antigens have also been found to be associated with an increased inflammatory response to COVID-19. The O blood group was found to be less susceptible to severe COVID-19 particularly compared to the A blood group [29–32]. This is probably correlated to the presence of serum autoantibodies to A and B antigens in O group patients that may decrease SARS-CoV-2 entrance into cells by binding to several components on the virus envelope. In addition, increased susceptibility of blood group A patients to severe COVID-19 may be correlated to increased ACE1 expression levels on lung epithelial cells [30].

Recent studies confirmed that the expression in the ABO gene locus (located in 9q34.2) of specific SNPs such as rs657152 and rs9411378 are respectively strongly and weakly associated to an increased risk of severe COVID-19 [33, 34].

Finally, the rs48697960 SNP (CT genotype) within the fucosyltransferase 2 (FUT2) gene which regulates expression of the ABO blood group antigens is weakly correlated with increased risk of severe COVID-19 development [13].

### Acquired risk factors for severe COVID-19

#### Aging

Age is a well-described risk factor for severe COVID-19 outcomes. Importantly, concomitant comorbidities and underlying genetic and acquired diseases need to be considered when interpreting age-related risk factors for severe COVID-19. Children are reported to be at similar risk of infection with SARS-CoV-2 as compared to other age groups, however are more likely to develop asymptomatic or mild SARS-CoV-2 infection, and are less likely to require hospitalisation or need of mechanical ventilation [35–37]. Children also develop robust cellular and humoral immune responses to SARS-CoV-2 which are sustained for > 12 months after infection, irrespective of COVID-19 severity [38]. Comparatively, increasing age is positively correlated with ICU admission, mechanical ventilation, and severe COVID-19 [39], with case fatality dramatically increasing from 50 years onwards [40, 41]. Severe pneumonia and acute lung injury were much more likely to occur in older patients [42], with reduced circulating lymphocytes and heightened circulating inflammatory biomarkers [40]. In line with human clinical observations, studies using golden hamsters have shown that older animals had dampened immune responses, impaired tissue repair responses and fewer germinal centre B cells resulting in lower neutralising antibody responses against SARS-CoV-2 infection [43].

Conversely, a study both ex vivo and in vitro has shown that SARS-CoV-2 infection is correlated with increased expression in alveolar cells type 2 of several cell senescence markers including p16^INK^. In addition, through a next-generation-sequencing analysis it was demonstrated that these senescent cells are correlated with increased risk to mediated SARS-CoV-2 mutations during its replication [44].

### Sex

Epidemiological studies have demonstrated that sex differences play a prominent role in determining severe COVID-19 and mortality outcomes. Males and females express varying levels of SARS-CoV-2 entry receptors such as ACE2 and transmembrane serine protease 2 (TMPRSS2), which is often further confounded by additional factors such as cigarette smoke (CS) exposure and increasing age [45]. Both males and females have been shown to be equally represented in terms of SARS-CoV-2 infection burden, irrespective of variable ACE2 expression [46]. Interestingly, males are much more likely to be admitted to intensive care unit (ICU) and have increased risk of mortality due to COVID-19 [46, 47]. Additional studies further support these findings, however they also reported that males were much more likely to have underlying chronic diseases such as systemic arterial hypertension, diabetes mellitus (DM), renal failure, and congestive heart failure, suggesting that confounding disease cannot be entirely excluded as an additional risk factor [48]. Comparison of SARS-CoV-2 viral burden in nasal swabs and saliva revealed no sex differences, however males were found to have significantly elevated CXCL8 and IL-18 pro-inflammatory mediators in plasma samples, likely contributing to severe COVID-19 and increased mortality [49].

### Cigarette smoke and chronic obstructive pulmonary disease

While, smoking habit seems not be correlated with increased mortality of COVID-19 patients, a study has shown that whilst smoking COVID-19 patients (n = 791) correlated with increased risk of complications, including c-ARDS and acute respiratory failure compared to never smokers COVID-19 patients (n = 5098), there were not differences in terms of mortality [50]. CS is the main aetiological factor for chronic obstructive pulmonary disease (COPD) development, which typically occurs after decades of chronic CS exposure [51]. COVID-19 diagnosis in COPD patients is often associated with increased hospital admission, mechanical ventilation, severe disease features and higher mortality [52], but there are contrasting data in literature. CS is known to upregulate ACE2 expression; the main SARS-CoV-2 viral entry receptor which is essential for infection and may ultimately predispose to elevated infection risk [53]. Recent work has shown that primary bronchial epithelial cells from COPD patients are much more susceptible to SARS-CoV-2 infection than healthy individuals, due to increased expression of SARS-CoV-2 entry receptors such as TMPRSS2 and cathepsin B, and that COPD cells had much higher pro-inflammatory cytokine responses and blunted type I IFN responses [54]. Moreover, another in vitro study analysing human bronchial epithelial cells, has confirmed the increased susceptibility to SARS-CoV-2 infection in COPD patients compared healthy subjects through the increased expression of the ACE2 receptor that mediate SARS-CoV-2 cell entry. In particular, the most susceptible cells to the SARS-CoV-2 entry were the globet cells that are increased in COPD patients [55]. COPD cells had 24-fold increased viral burden compared to healthy subjects, reiterating the importance of heightened SARS-CoV-2 co-receptor expression in facilitating infection [54]. Works from in vivo animal models is eagerly awaited.

### Diabetes mellitus

DM is the most important risk factor for developing severe COVID-19 [56]. There are many studies that show increased risk of development severe COVID-19 in patients with DM compared to COVID-19 patients without this disease; in addition, several evidence show how COVID-19 mortality is negatively associated with glycaemic control both before and during the hospital stay. Moreover, higher serum levels of HbA1c are also positively correlated with an increased risk of mortality from COVID-19 patients with DM [57–60]. Patients with DM have an increased expression of ACE2 in both lungs and endothelium that can increase chronic inflammation, impair the endothelial cell activation, enhance the pro-inflammatory response, and therefore determine a dysfunction of the alveolar-capillary barrier [61, 62].

### Obesity

Obesity is another risk factor for hospitalization rate and ICU admission due to COVID-19 and a body mass index (BMI) > 40 has been found to be an independent risk factor for mortality in COVID-19 patients [63–66]. Obese adipocytes express high levels of ACE2 receptor, may increase IL-6 circulating levels and induce a pro-thrombotic state. Moreover, impaired chest-wall elastance and decreased lung function can contribute to acute respiratory failure [67, 68].

Conversely, a retrospective study on German hospitalized COVID-19 patients has shown that obesity and in particular grade I obesity, did not increase the patient mortality [69].

### Systemic arterial hypertension

Systemic arterial hypertension increases the risk of severe COVID-19, need of invasive mechanical ventilation and death [70–72] and this can be related to an unbalance of the renin–angiotensin–aldosterone system. In addition, patients with systemic arterial hypertension treated with ACE2 receptor blockers showed a decreased mortality due SARS-CoV-2 infection compared to untreated patients with uncontrolled blood pressure values or treated with other forms of anti-hypertension medications [56].

### Chronic kidney diseases

Chronic kidney diseases also represent an important risk factor for severe COVID-19, hospitalization, and mortality, but the molecular mechanism that induce increased COVID-19 severity mediated by chronic kidney diseases are still not clear [73].

### Idiopathic pulmonary fibrosis

Idiopathic pulmonary fibrosis (IPF) is a well-known risk factor for severe COVID-19 outcomes and increased mortality as compared to the general population [52, 74] but there are contrasting data in the literature. IPF is also known to increase the risk for hospitalisation, critical care, acute kidney injury and need for mechanical ventilation in COVID-19 patients [75]. IPF and COVID-19 share similarities in disease features, such as stiffening of pulmonary tissue and impaired gas exchange [76]. To date, there has been no laboratory study which has investigated the causal relationship between IPF and severe COVID-19.

### Protective genetic factors for severe COVID-19

As well as genetic susceptibility, several data demonstrated several factors correlated with decreasing risk of SARS-CoV-2 infection and with the development of severe disease COVID-19 and these are summarized in Table 1.

### TMPRSS2 SNPs

The SNP rs12329760 within the TMPRSS2 gene is correlated with decreased susceptibility to COVID-19 when the T allele is expressed as the C/T genotype [31, 77], and particularly as the T/T genotype. The latter genotype being characterized by further decreased susceptibility to COVID-19 [78]. The rs12329760 SNP also correlated with a decreased risk of development of severe COVID-19, mediated through a decreased expression of the receptor for SARS-CoV-2 along with a decreased affinity binding to SARS-CoV-2 [7].

### HLA system SNPs and alleles

Despite there being very little data about class I and II HLA alleles correlated to decreased risk to development of COVID-19, a study has shown that the alleles HLA-A*02:01 and HLA-A*03:01, only when express in homozygosity, are not correlated to severe COVID-19 development [23]; HLA-A*68:02 was correlated to decreased risk to ICU admission in hospitalized COVID-19 patients and in addition, HLA-B*14 was correlated to decreased mortality in COVID-19 patients [27], however, the molecular mechanism is still not fully clear.

### Protective pro-inflammatory mediators signalling

A study evaluating a Spanish population has shown that the deletion of the CCR5 variation Δ32 (rs333), (in homozygosis genotype) is correlated to mild disease induced by SARS-CoV-2 infection [79].

### Immune system dysregulation in the COVID-19 pathogenesis

**Fig. 1:**
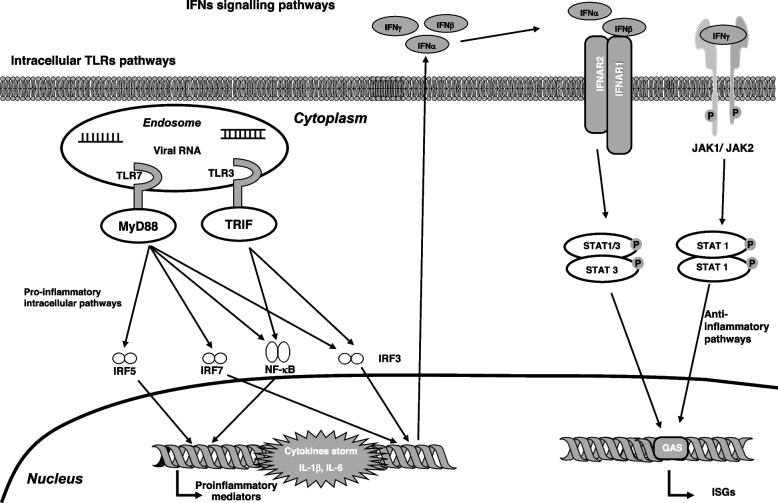
IFNs signalling pathway activation mediated by intracellular TLRs following viral infection. Several intracellular TLRs such as TLR-3 and -7 can recognize viral RNA of SARS-CoV-2, activating several intracellular pro-inflammatory pathways including NF-κB and inducing transcription and release of several pro-inflammatory mediators (including IL-1β and IL-6) forming the so termed “cytokines storm”. Beetwen these mediators, also anti-viral cytokines such as IFNα, IFNβ, IFNγ can be released. The mediators IFNAR1, -2 and JAK1, -2, respectively can activate intracellular anti-inflammatory pathways involving STAT1, -2, mediating expression of ISGs. Abbreviations: GAS: interferon-gamma activated sequence; IFN: interferon; IFNAR: interferon-alpha, -beta, and -omega receptor; IRF: IFN regulatory factor; ISGs: interferon-stimulated gene; JAK: janus-kinase; MyD88: myeloid differentiation primary response gene 88; NF-κB: nuclear factor kappa B; P:phosphate; RNA: ribonucleic acid; STAT: signal transducer and activator of transcription; TLR: toll-like receptor; TRIF: TIR domain containing adapter inducing interferon β

Growing evidence shows that the development of severe COVID-19 and of c-ARDS are determined by the dysregulation of many components of the innate immune system, including alveolar macrophages, blood monocytes, neutrophils and complement [80, 81]. While, further data are needed to better clarify the role of adaptative immunity in the pathogenesis of COVID-19 and c-ARDS, several lines of evidence show that the three main components of the adaptive immune system, such as CD4+ T cells, CD8+ T cells, and B cells producing neutralizing antibodies, all contribute to controlling SARS-CoV-2 infection during the acute phase and regulate immune memory [1, 81]. Dysregulation of these components can induce a more severe clinical presentation [82]. In addition, severe COVID-19 patients exhibit a correlation to increased expression of several genes mediating activation of inflammatory innate immune responses compared to both moderate and mild COVID-19 patients [83]. This includes activation of: intracellular IFN and TLR signalling pathways (Fig. 1), signal transducer and activator of transcription (STAT)3 and receptors expressed on myeloid cells (TREM1) to CXCL8, IL-6 and IL-22.

### Blood

Blood neutrophils of COVID-19 patients are represented by a particular subpopulation characterized by enhanced expression of several gene correlated to IFNs pathways including interferon inducing protein (IFI)44L, IFI44, interferon-induced protein with tetratricopeptide repeats (IFITINIB1) and IFI6 when compared to blood neutrophils of healthy subjects, but also when compared to patients with bacterial ARDS [84].

A study has shown that blood neutrophils of severe/critical COVID-19 patients (*n* = 44), were characterized by several markers of immature and immunosuppressive state including decreased surface CD13 expression and increased surface CCR5 expression, compared to blood neutrophils of patients with mild SARS-CoV-2 infection (*n* = 40) and to non-infected subjects (*n* = 22) [85].

Similarly, another study has shown that COVID-19 patients (*n* = 27) are characterized by blood neutrophils expressing increased levels of neutrophil activation markers, such as CD11b and CD66b compared to healthy subjects (*n* = 12). In addition, these neutrophils also expressed increased levels of CD47 that positively correlated to enhanced neutrophils survival [86].

In addition, blood neutrophils of COVID-19 patients (*n *= 15) when stimulated with phorbol 12-myristate 13-acetate (PMA) produced increased levels of reactive oxygen species (ROS) when compared with healthy subjects; in addition, neutrophil ROS production was positively correlated with blood neutrophil levels in COVID-19 patients. However, there was no difference in neutrophil ROS production when comparing neutrophils of COVID-19 patients against patients with sepsis unrelated to SARS-CoV-2 infection [87].

Whilst no significant differences were found in blood monocytes between severe (*n* = 43), moderate (*n* = 16) and mild (*n *= 27) COVID-19 patients, severe patients did show a blood monocyte sub-population that mostly expressed decreased “nonclassical” CD14^Low^CD16^High^ markers, whereas monocytes from mild/moderate patients expressed enhanced CD14^High^CD16^High^ markers. In addition, severe COVID-19 patients showed increased blood neutrophil levels expressing an immature phenotype characterized by CD10^Low^CD101- and elevated release of calcium binding protein (S100) A8/9 [88]. Moreover, COVID-19 patients may have a reduced number of circulating monocytes levels [89]. Profiling of peripheral blood mononuclear cells from COVID-19 patients (*n* = 125), mild infected SARS-CoV-2 patients (*n *= 36) and healthy subjects (*n* = 60) helped to identify three different immunotypes correlated with disease severity. The phenotype that correlated with the most severe disease was characterized by highly activated CD4+ and CD8+ T-cells and enhanced plasmablast responses. The second phenotype that correlated less with disease severity was characterized by decreased CD4+ T cell activation and enhanced proliferation of B memory cells. The third phenotype was unrelated to severe disease and was characterized by a low or an absence T and B cell activation [90].

One of the first studies on the role of the adaptative immune system in COVID-19 showed reduced blood levels of both CD4+ and CD8+ T cells in COVID-19 patients (*n* = 522) compared to healthy subjects (*n* = 40) [91]. Blood total T cells levels negatively correlated with severity and mortality in COVID-19 patients and those admitted to ICU (*n* = 43) had further decreased total blood T cells levels compared to COVID-19 patients not admitted to ICU (*n* = 479). Another study confirmed these findings where blood levels of both CD4+ and CD8+ T cells from severe COVID-19 patients (*n* = 11) were decreased compared to moderate COVID-19 patients (*n* = 10) [92]. These findings correlated with decreased serum IFN levels produced by CD4+ T cells.

Aging is a well-known important risk factor for increased severity of COVID-19 and is characterized by changes in adaptative immunity with a decreased T cell response to viral infection, a decreased generation of CD3+ and naïve T cells, inversion of blood CD4+:CD8+ T cell ratio through decreasing blood CD8+ T cells, and enhanced proliferation of regulatory T cells (Treg) [93] that are immune-suppressive cells. Interestingly, a cohort study of 12 hospitalized COVID-19 patients showed than an enhanced and rapid blood T cell activation and response against SARS-CoV-2 infection was correlated with reduced disease severity and length of hospitalization [94].

Moreover, a recent study has shown that aging can alter subset clustering of both B and T cells, in particular, hospitalized COVID-19 patients > 70 years of age (n = 10), when compared to hospitalized COVID-19 patients < 60 years of age (n = 7), showed decreased blood levels of both CD8 + naïve T cells (CD8+, CCR7+, CD45RA+, CD45R0-, CD27+, CD28+) and CD4 + memory T cells (CD4+, CCR7+, CD54RA-, CD45R0+, CD27+, CD28+), these were also characterized by a decreased proliferation index. Conversely, hospitalized COVID-19 patients > 70 years of age showed increased blood plasmablast B cells (CD19^low^, CD20-, IgD-, IgM-, CD80+, CD38+), compared to hospitalized COVID-19 patients < 60 years of age [95].

### Serum biomarkers

Patients with prominent B cell responses had increased severity and risk of mortality. In particular, in paediatric COVID-19 patients, who are typically characterized by better prognosis compared to adults/older patients, both severe (*n *= 16) and non-severe (*n* = 31) were characterized by decreased B cell response compared to both severe (*n* = 19) and non-severe (*n* = 13) adult patients, showing both decreased serum levels of antibodies against SARS-CoV-2, but in addition different antibodies class and type, where paediatric patients showed mainly production of IgG against Spike protein, while adult patients were characterized by production of IgG, IgM and IgA against both Spike protein and nucleocapsid proteins [96].

Moreover, hospitalized COVID-19 patients (*n* = 376) had increased serum levels of both IgG and IgA anti-Spike and anti-nucleocapsid antibodies levels compared to asymptomatic SARS-CoV-2 infected patients [97]. In addition, IgG and IgA anti-Spike and anti-nucleocapsid antibodies levels positively correlated with COVID-19 severity. The subtypes of the antibodies are critical and the antibodies able to targeting the class 4 epitope, can bind the conserved receptor binding domain supersite and sterically inhibit ACE2 interactions are most effective [98].

Finally, several data have shown that in hospitalized COVID-19 patients, but not in asymptomatic/mild SARS-CoV-2 patients, the presence and increased levels of one or more serum auto-antibodies [including anti-phosphatidylserine/prothrombin (aPS/PT) IgM and IgG, anticardiolipin IgM and IgA, and anti-β2-glycoprotein 1 IgG positively correlated with increased severity of COVID-19 mortality [99–101], and, in vitro, to increased neutrophil extracellular traps (NETs) [101].

### Bronchoalveolar lavage biomarkers

Bronchoalveolar lavage (BAL) in severe/critical COVID-19 patients (*n* = 28) is characterized by increased neutrophil levels and decreased lymphocytes and alveolar macrophages levels, compared to mild/moderate COVID-19 patients (*n* = 5) [102]. In addition, BAL alveolar macrophages from severe/critical COVID-19 patients showed an increased surface expression of several markers, such as ficolin-1 and secreted phosphoprotein 1 (SPP1). Moreover, the increased expression of fatty acid-binding protein 4 (FABP4) correlated with mild/moderate COVID-19 disease [103].

### Lung tissue

Analysis of postmortem lung biopsies taken from 7 patients who died from COVID-19 has shown that the main immune cells localized in alveolar space of all these patients were represented by alveolar macrophages and T-cell lymphocytes. Interestingly, biopsy samples from 3 of these patients were also characterized by increased localization of neutrophils in the alveolar space [104].

Additional studies on patients who died following COVID-19 infection also showed that lung tissue was characterized by an increased infiltration of alveolar macrophages [105]. Conversely other analysis have found an increased infiltration of CD8 + T-lymphocytes [106].

Finally, more recent and robust data focusing immune inflammatory cells expressed in post-mortem lung tissue of COVID-19 patients has confirmed that levels of several immune cells can change between COVID-19 patients. However, the immune cell populations that have the greatest increase and prevalence in the interstitial space and alveolar space are represented by CD3+, CD4+, CD8+ and CD45+ lymphocytes and macrophages [107]. In particular, lung tissue levels of macrophages and CD8+ T-cells positively correlated with diffuse alveolar damage (DAD) in COVID-19 patients who had died from the disease [108].

### Pro-inflammatory signalling pathways in COVID-19 pathogenesis

#### Blood

As shown previously, severe COVID-19 patients are characterized by increased blood neutrophil levels. Additional studies have confirmed this correlation and in addition shown that in severe COVID-19, blood neutrophils are characterized by an altered expression though epigenic control (hyper- or hypomethylation) of several genes correlated to viral defense such as; type I IFNs, 2-prime,5-prime oligoadenylate synthetases (OAS)1, -2, IFIT3, IRF7, TRIM2 [109].

Patients with severe/critical COVID-19 were also characterized by an increased activation of TLR2 pathway with enhanced expression of TLR2 RNA in the circulating mononuclear cells, when compared with moderate COVID-19 or healthy subjects. In addition, these data were confirmed in vivo since mice treated with a TLR2 inhibitor did not develop severe disease after SARS-CoV-2 infection [110].

A study by Kunadi et al. showed that blood CD8+ T cells from infected SARS-CoV-2 patients (*n* = 17 non hospitalized, *n* = 13 hospitalized non-ICU, *n* = 9 ICU patients) had different grades of activation that positively correlated with disease severity [111]. The most active CD8+ T cells had impaired IFN-responses and cytotoxicity, expressed CD38, CD279 and HLADR and a cluster of several pro-inflammatory genes characterized by increased release of mediators such as; tumour necrosis factor (TNF)α, IFNγ, CCL3, CCL4, CXCL1 and CXCL2 [9].

### Serum pro-inflammatory mediators

Serum NETs levels are increased in SARS-CoV-2 infected patients (*n* = 33) as compared to healthy controls (*n* = 17) and their levels positively correlated with thrombosis risk and disease severity, since higher levels were found in patients intubated or that died [112].

In COVID-19, increased levels of IL-6 support the differentiation of pro-inflammatory Th17 cells and a decrease of the T cells, such as CD4+ T cells, CD8+ T cells and natural killer (NK) cells [113]. This induced lymphopenia prevents the production of antiviral antibodies by the adaptive immune system, and delays or precludes the clearance of the virus. Literature data also shows that complement pathway is enhancing activated in SARS-CoV-2 infection and positively correlated with disease severity [80].

Serum C5a and C5aR levels were increased in COVID-19 patients (*n* = 82), compared to healthy subjects (*n* = 10) and C5a/C5aR serum levels positively correlated with disease severity where the most severe COVID-19 patients (*n* = 28) showed the most increased levels compared to moderate (*n* = 34) and mild (*n* = 10) patients [114].

Serum Cb5 levels were also increased in COVID-19 patients (n = 134), compared to hospitalized patients with influenza (*n* = 54). Moreover, further increased serum Cb5 levels were found in ICU COVID-19 patients (*n* = 72) compared to non-ICU COVID-19 patients (*n* = 62) and positively correlated with increased mortality risk [115]. Similarly, another study showed that serum levels of several complement subunits such as C5b, C5a, C3bc and C4d were increased in hospitalized COVID-19 patients (*n* = 39) compared to healthy subjects. However, serum C5b and C4d levels were further increased or tended to increase during hospitalization in COVID-19 patients with acute respiratory failure (*n* = 23) [116]. Recently, increased complement levels and activation (C3a fragment) were found in the lung tissue of critically ill (*n *= 24), compared to non-critically ill (*n* = 49), COVID-19 patients [117].

### BAL pro-inflammatory mediators

BAL alveolar macrophages from severe COVID-19 patients (*n* = 23) were characterized by an increased production and release of several pro-inflammatory mediators, such as IL-1β, IL-6, CCL3 and CCL4 and increased expression of IL-1R and S100A8/9 proteins, compared to those from mild COVID-19 patients [118].

In addition, severe COVID-19 patients BAL alveolar macrophages (*n* = 88) are characterized by a cluster activation, involving genes encoding several pro-inflammatory mediators such as CCL7, CCL8 and CCL13, that can mediate recruitment and activation of monocytes and T cells [119].

An alveolar macrophage sub-population characterized by increased secretion of CCL8, CXCL10, CXCL11, and IL-6 and a blood monocyte sub-population characterized by increased secretion of IL-1β, CCL20, CXCL2, CXCL3, CCL3, CCL4, and TNFα were found in the BAL of severe COVID-19 patients (*n* = 38) or patients that were characterized by a severe progression of COVID-19 (*n* = 54) [120].

In a cohort study of severe COVID-19 patients (*n* = 9) it was shown that their BAL contained a clonal expansion of a sub-population of memory Th17 cells that increase the secretion of several pro-inflammatory mediators, such as IL-17A and granulocyte–macrophage colony-stimulating factor (GM-CSF) [121].

### Lung tissue pro-inflammatory biomarkers

Analysis of postmortem lung tissue taken from patients who died from COVID-19 showed that SARS-CoV-2 infection caused diffuse alveolar damage (DAD) characterized by formation of hyaline membranes in lung parenchyma and perivascular lung vessels inflammation inducing intravasal fibrin thrombi/microthrombi [122–124]. Moreover, DAD extension was positively correlated with male sex and premortem serum LDH levels [106], while large vessels thrombosis and microthrombi of lung vessels were found in deceased COVID-19 patients who had a longer average disease duration compared to COVID-19 patients who died after a short disease duration [125].

Furthermore, deceased COVID-19 patients had increased infiltration of alveolar macrophages into the lung along with increased expression of several pro-inflammatory mediators such as IL-6 [105].

Similar to “classical” ARDS [126], c-ARDS is characterized by a specific pro-inflammatory mediator profile in several lung compartments that represent promising biomarkers for diagnosis, stratification of disease severity, prognosis and response to therapy.

### Lung epithelial and alveolar cell biomarkers in c-ARDS

Different lung epithelial and alveolar biomarkers have been found in COVID-19 patients, which could help diagnosis and monitoring of patients [127, 128]. Growing evidence shows that several proteins expressed by the epithelial cells of the lower airways in COVID-19 correlate with acute lung injury and potential fibrotic evolution of both lung epithelial and alveolar cells. These include Krebs von den Lungen-6 (KL-6), surfactant proteins (SP-A, SP-B, SP-C, SP-D), and club cell secretory protein 16 (CC16) [127], representing potential biomarkers in acute SARS-CoV-2 pulmonary infection [129].

### Krebs von den Lungen-6

Serum KL-6 levels are increased during severe, but not in mild, SARS-CoV-2 infection. This can be explained by epithelial cell injuries that can induce leakage in the air-blood barrier, increasing serum levels of KL-6 [127]. Thus, KL-6 represents a potential biomarker of severe cases and correlates with fibrotic evolution of lung parenchyma but has low sensitivity [130]. Indeed, increased serum KL-6 levels were found in severe COVID-19 (*n* = 12) compared to mild SARS-CoV-2 infected patients (*n* = 10) [131]. Another study confirmed increased serum KL-6 levels in COVID-19 patients (*n *= 21) compared to healthy subjects (*n* = 43). In addition, serum KL-6 levels positively correlated with disease severity in terms of decreasing oxygen partial pressure (PaO2) and SARS-CoV-2 induced pneumonia as assessed by chest CT scan [132].

In particular, the presence and extension of lung opacity at lung CT scan in SARS-CoV-2 infected patients correlated with increased KL-6 levels compared to SARS-CoV-2 infected patients without lung opacity [133]. Interestingly, serum KL-6 levels were increased in COVID-19 patients (*n* = 83) compared to healthy subjects (*n* = 70), positively correlating with severity, but not when compared with serum levels in patients with other lung interstitial diseases (*n* = 31) including IPF and sarcoidosis [134]. Moreover, increased serum KL-6 levels have been found in severe COVID-19 patients (*n* = 21) as compared to non-severe ones (*n* = 54) and serum KL-6 levels further increased after 1 week from diagnosis in severe patients [135]. Similarly, although serum KL-6 levels were increased in hospitalized COVID-19 patients with SARS-CoV-2-mediated pulmonary fibrosis (*n* = 12) compared to hospitalized COVID-19 patients without pulmonary fibrosis (*n* = 10), serum KL-6 levels tended to decrease after hospital discharge in a time-dependent manner correlating with the resolution of pulmonary fibrosis [131]. Another study confirmed increased serum KL-6 levels in severe COVID-19 patients (*n* = 36), compared to moderate (*n* = 28) and mild (*n* = 49) patients, demonstrating that serum KL-6 levels positively correlated with an increased risk of developing lung fibrosis [136].

### Surfactant proteins

Many studies have evaluated levels of surfactant proteins (SP) in serum during both acute pulmonary diseases (such as “classical” ARDS) and chronic lung diseases (such as COPD and IPF) [127]. Growing evidence shows that serum SPs levels are increased in COVID-19 patients and correlate with disease severity, suggesting that SPs may be useful biomarkers in COVID-19. In particular, SP-D seems to have a key role in pathogenesis of c-ARDS correlating with COVID-19 severity [137]. Studies measuring SP-D after SARS-CoV-1 and SARS-CoV-2 infections found higher levels in both disease and correlated with the disease severity [138]. Moreover, SP-D serum levels are higher in COVID-19 patients (*n* = 88) as compared to healthy subjects (*n* = 20) and are furtherly increased in those with poor prognosis (*n* = 33) [139]. Finally, due to the key role of SPs in pathogenesis of severe COVID-19, use of systemic or inhaled exogenous SPs as therapeutic options to prevent the most severe COVID-19 form has been proposed and currently there are several promising data in vivo evaluating effectiveness of these compounds [140].

### Club cell secretory protein 16

There are several studies showing that serum CC16 levels are positively correlated to inflammatory damage impairing the alveolar-capillary barrier especially when induced by acute lung injury (ALI) or “classical ARDS” representing a biomarker correlated to “classical ARDS” severity [127, 141, 142]. Although data on the role of CC16 in COVID-19 and c-ARDS are poor, serum CC16 protein levels are increased in COVID-19 and c-ARDS [143].

### Soluble receptor for advanced glycation end products

Levels of the soluble receptor for advanced glycation end products (sRAGE) in serum are increased in several inflammatory disease associated to lung epithelial injury, such as bacterial infection, COPD, lung fibrosis and “classical” ARDS [126, 144]. However, there is limited data on the role of sRAGE in c-ARDS. One study showed that serum sRAGE levels are increased during SARS-CoV-2 infection (*n* = 164) compared to healthy subjects (*n* = 15), and positively correlated with COVID-19 severity, the need for mechanical ventilation and/or high flow oxygen therapy and increased mortality [145]. However, there were no significant differences between COVID-19 patients and patients with pneumonia unrelated to SARS-CoV-2 infection (*n *= 23). Similarly, another study showed that increases in serum sRAGE levels in COVID-19 patients during hospitalization correlated with increased risk of ICU admission and mortality [146]. Conversely, another study showed increased serum sRAGE levels in asymptomatic SARS-CoV-2 infected patients (*n* = 23) compared to COVID-19 patients with pneumonia (*n *= 35) [147].

Finally, a recent study showed that serum sRAGE levels were increased in severe COVID-19 patients in need of mechanical ventilation (*n* = 73) compared to those who did not (*n* = 152). In addition, sRAGE levels tended to decrease during hospitalization and could potentially be an early biomarker of COVID-19 severity [148].

### Lung endothelial cell biomarkers in c-ARDS

As previously reported, in COVID-19 patients, the pathogenesis of disease is related to immune responses to SARS-CoV-2, which is responsible for direct tissue damage, with the interplay of the endothelium, complement activation and pro-coagulable states playing a significant role during the later stages [149].

Moreover, increased data obtained through several post-mortem studies analysing lung tissue of deceased patients showed that SARS-CoV-2 infection is correlated to coagulopathy inducing the formation of microthrombi in lung tissue as well as in extra-pulmonary organs [107, 150].

Similar to “classical” ARDS [126] there are several molecules that have been identified as endothelial dysfunction biomarkers in c-ARDS, such as ACE2 receptor, von Willebrand factor (vWF), angiopoietin-2 (Ang2) and plasminogen activator inhibitor (PAI)-1. These tend to be increased in the serum of COVID-19 patients compared to healthy subjects and correlate with disease severity and mortality [151].

**Fig. 2:**
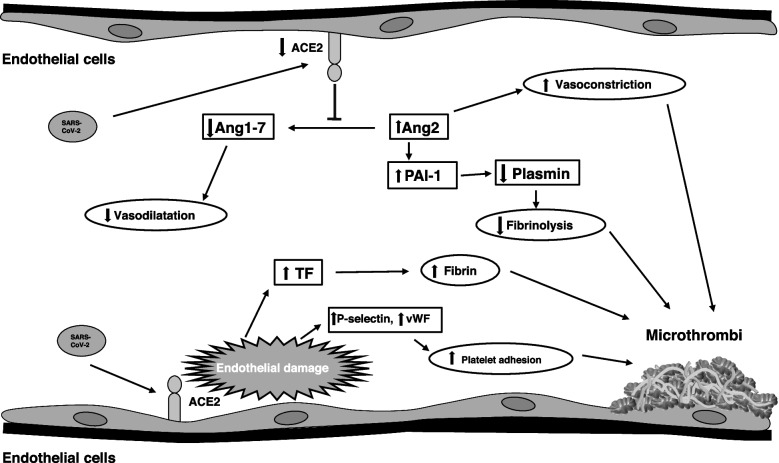
Pro-thrombotic status induced by SARS-CoV-2 infection. SARS-CoV-2 can bind endothelial ACE2 receptor inducing downregulation. Decreased levels of ACE2 induce decreased levels of Ang1 and -7 and consequently vasoconstriction. Moreover, Ang2 tends to accumulate inducing vasoconstriction and increased levels of PAI-1 and thus, decreased fibrinolysis. Conversely, endothelial damage SARS-CoV-2 induced, mediate increased levels of TF inducing increased fibrin levels and increased levels of vWF and P-selectin mediating enhanced platelet adhesion. Abbreviations: ACE: angiotensin-converting enzyme; Ang: angiopoietin; PAI-1: plasminogen activator inhibitor-1; TF: tissue factor; vWF: Von Willebrand factor

In particular, SARS-CoV-2 entry into endothelial cells induces a prothrombotic status through the decreased levels of ACE2 and the increased release of coagulation factors such as tissue factor (TF), Ang2, fibrin and increased platelet adhesion mediated by direct virus-induced endothelial damage. In addition, accumulation of PAI-1 and Ang2 decreases fibrinolysis activity which in turn mediates a vasoconstriction effect [152] (Fig. 2).

Thus, these molecules may be used to identify patients with risk factors for c-ARDS and enable better clinical stratification following diagnosis, considering that endothelial biomarkers are significantly higher in patients requiring high-flow oxygen or mechanical ventilation at ICU admission [153]. In addition, growing data shows that endothelial dysfunction during COVID-19 has a key role in the pathogenesis of several thrombotic complications such as myocardial infarction, stroke and pulmonary embolism [151, 154]. Biomarker levels of endothelial injury such as Ang2 are increased in c-ARDS, probably because endothelial damage may be predominant in the pathogenesis of severe forms of ARDS [155]. Consequently, defining the molecular mechanisms of endothelial dysfunction, could have diagnostic, prognostic and therapeutic implications [156].

### ACE2 receptor

Growing evidence shows that a correlation exists between SARS-CoV-2 endothelial damage, clinical manifestations, and organ failure, reflecting the impact of high immune inflammatory responses [156, 157], although some studies found similar ACE2 levels in SARS-CoV-2 infected patients (*n* = 24) and healthy subjects (*n* = 61) [158].

Both lung tissue and serum ACE2 levels were increased in severe COVID-19 patients with c-ARDS (*n* = 15) compared to healthy subjects (*n* = 13). However, both ACE2 levels were also increased in patients with “classical” ARDS, showing no significant differences with COVID-19 patients [159]. Another study showed that serum ACE2 levels were increased in COVID-19 patients with c-ARDS (*n* = 68) compared to those without c-ARDS (*n* = 21) [160]. Similarly, serum ACE2 levels were increased in critical (*n* = 110; mean SOFA score 11) compared to non-critical (*n* = 66, mean SOFA score 4) COVID-19 patients, but also patients with severe sepsis (*n* = 32). In addition, increased serum ACE2 levels were found in critical COVID-19 patients who died (*n* = 86) compared to survivors (*n* = 24) [161].

Moreover, autoantibodies against ACE2 positively correlated with severity of SARS-CoV-2 infection and they were higher in moderate (*n* = 68) compared to mild (*n* = 31) COVID-19 patients, and were further increased in severe COVID-19 patients (*n* = 20) [162].

### Von Willebrand factor

In “classical” ARDS, serum vWF levels are increased and positively correlate with risk of mortality [126]. Growing evidence shows that both vWF and thrombospondin type 1 motif member (ADAMTS)13 have a key role in thrombus formation and organ failure in COVID-19 patients [163]. Decreased serum ADAMTS13 levels correlate with increased hospitalization risk in COVID-19 patients and poor outcomes [149]. Serum vWF levels and ADAMTS13 activity are positively and negatively correlated with COVID-19 severity, respectively. Severe COVID-19 patients (needed for mechanical ventilation, *n* = 19) showed the greatest increase in serum vWF levels and decreases in ADAMTS13 activity compared to moderate (needed for high flow oxygen therapy, *n* = 17) and mild (needed for low-flow oxygen therapy, *n* = 14) patients [164]. Similarly, serum vWF levels in COVID-19 patients (*n* = 102) compared to healthy subjects (*n* = 26) is associated with severity and ICU admission (*n* = 17). Moreover, deceased COVID-19 patients (*n* = 25) showed the greatest increase in serum vWF levels, as compared to hospitalized COVID-19 patients with (*n* = 33) and without oxygen therapy (*n* = 27). In addition, serum ADAMTS13 activity tended to negatively correlate with COVID-19 severity, with the greatest decreases in serum ADAMTS13 activity being found in deceased patients [165].

These findings were confirmed by another study showing that serum vWF levels were increased in COVID-19 patients admitted to ICU (*n* = 32) compared to non-ICU COVID-19 patients (*n* = 32), whereas serum ADAMTS13 levels were decreased and negatively correlated with patient mortality and SOFA score [166].

Nevertheless, Doevelaar et al. did not find any difference in ADAMTS13 levels between healthy subjects (*n* = 30) and COVID-19 patients (*n* = 75). However, the ADAMST13/vWF ratio tended to decrease in COVID-19 patients compared to healthy controls, negatively correlating with severity disease [167]. Levels of both serum vWF and its multimers were increased in SARS-CoV-2 infected patients (*n* = 127) compared to healthy subjects (*n* = 23). Moreover, these levels positively correlated with COVID-19 severity where critical COVID-19 patients (*n* = 89) had the most increased levels compared to non-critical (*n* = 96) and outpatients (*n* = 23) COVID-19 patients [168].

Another study showed that serum vWF levels in severe COVID-19 patients admitted to ICU (*n* = 28) tended to increase during the first days of hospitalization and to then decrease after the acute phase, until 3 months of convalescence following ICU discharge. However, severe COVID-19 patients who died in ICU (*n* = 3) had significantly higher vWF levels at ICU admission that did not decrease during hospitalization, although these findings were based on low patient numbers [169].

Serum vWF levels at the time of hospital admission correlates with different grades of COVID-19 severity (mild, moderate and severe/critical; *n* = 333), and with increased risk of mortality during hospital stay [170]. These findings were confirmed also by Thomas et al. who found serum vWF levels being correlated with COVID-19 severity showing different levels in severe (*n* = 36), moderate (*n* = 36) and mild (*n* = 34) COVID-19 patients [171], with vWF levels independently associated with mortality. Finally, a study performed on lung tissue of deceased COVID-19 patients (*n* = 9) also found increased tissue expression of vWF [172].

### Plasminogen activator inhibitor-1

Serum PAI-1 levels positively correlate with poor prognosis and mortality in ALI/ “classical” ARDS [126]. However, currently there is limited data on the role of PAI-1 in the pathogenesis of c-ARDS. One study showed that serum PAI-1 levels were increased in COVID-19 patients admitted to the hospital compared to less severe COVID-19 patients, and levels positively correlated with mortality [173].

### Endothelial cell adhesion molecules

Increased levels of vascular cell adhesion molecule (VCAM)-1, ICAM-1, P-selectin and E-selectin were found in lung oedema and serum from patients with “classical” ARDS. Although these molecules seem to have a role in the pathogenesis of COVID-19 correlating with disease severity and thrombosis development, their pathways of involvement are still unclear [174]. First reports have shown that serum VCAM-1 and ICAM-1 levels were increased in COVID-19 patients (*n* = 36), as compared to healthy subjects (*n* = 16), positively correlating with disease severity and tending to decrease during convalescence [175]. However, although another study confirmed the increased levels of VCAM-1 in COVID-19 patients (*n* = 14) compared to healthy subjects (*n* = 14), others adhesion molecules such as ICAM-1 and P-selectin did not differ between these groups [176]. Another study showed that in the serum of COVID-19 patients (*n* = 250) VCAM-1 and ICAM-1 levels positively correlated with SARS-CoV-2 RNAemia. Moreover, increasing serum VCAM-1 levels in COVID-19 patients during hospitalization correlated with increased risk of ICU admission and mortality [146]. In severe COVID-19 patients admitted to ICU (*n* = 38), increased levels of ICAM-1 and E-selectin, but not VCAM-1 or P-selectin, were found in COVID-19 patients who died during hospitalization (*n* = 10), compared to survivors (*n* = 28) [160]. Both P- and E-selectin levels were increased in the serum of COVID-19 patients (*n* = 103) compared to healthy controls, and a further increase was found in those who developed thrombosis (*n* = 35) compared to those who did not (*n *= 68). Moreover, increased serum sVCAM-1 levels were independently associated with longer duration of mechanical ventilation [177]. In contrast, a study of lung tissue from patients who died from COVID-19 (*n* = 9) showed a reduced local expression of ICAM-1, VCAM-1, E-selectin, and P-selectin [172].

### Neo-angiogenic proteins

Vascular endothelial growth factor (VEGF) and Ang2 drive neo-angiogenesis and vascular formation, are upregulated during inflammatory responses, and correlate with mortality in both COVID-19 and “classical” ARDS [178]. Several studies showed how VEGF/Ang2 axis is involved in both “classical” ARDS and c-ARDS pathogenesis [126, 179]. These data show that increased serum levels of both VEGF and Ang2 positively correlate with increased ARDS severity and risk of mortality [126]. Serum Ang2 levels were also increased in severe COVID-19 patients needing mechanical ventilation (*n* = 73) as compared to patients that did not need it (*n* = 152). However, increases in Ang2 levels started 3 days after hospitalization representing a late biomarker of COVID-19 severity [148]. Moreover, increased serum levels of Ang2 were found in patients with c-ARDS (*n *= 31) compared to hospitalized COVID-19 patients without ARDS (*n* = 12); serum Ang2 levels were positively correlated to severity of disease and mortality. In addition, lung autopsy of deceased patients showed that Ang2 levels correlated with microthrombi development and necrotic lung endothelial cell death [180].

### Serum pro-inflammatory mediators as biomarkers of c-ARDS

**Fig. 3:**
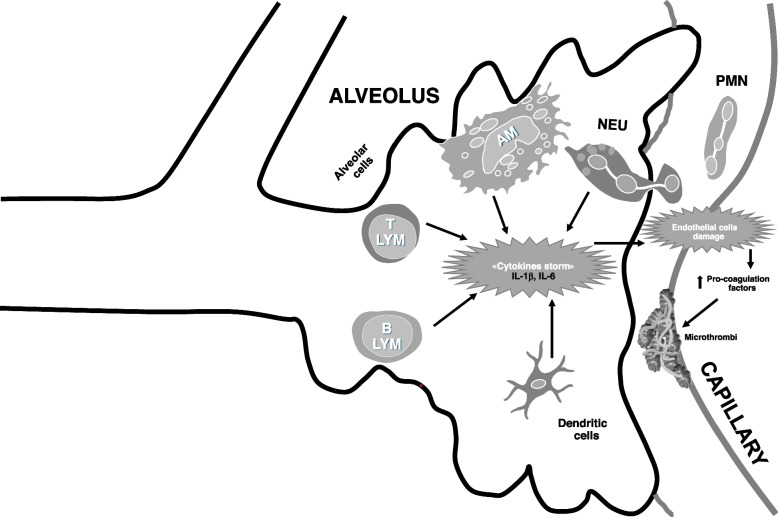
Immune cells involved in the development of a “cytokines storm” and severe COVID-19. Both innate and adaptative immune cells are involved in secretion of several pro-inflammatory mediators (including IL-1β and IL-6) forming the so termed “cytokines storm”. This induces alveolar damage, vascular endothelial damage and increased pro-thrombotic factors mediating micro-thrombi development. On the other hand, the “cytokine storm” can induce recruitment and activation of others lung immune cells maintaining the pro-inflammatory status. Abbreviations: AM: Alveolar macrophage; IL: interleukin; LYM: lymphocyte; NEU: neutrophil; PMN: polymorphonuclear

The hallmark of COVID-19 pathogenesis is an unrestrained and abnormal over-production of pro-inflammatory mediators. This so-called “cytokine storm” (Fig. 3) is characterized by increased levels of cytokines and chemokines including GM-CSF, IL-2, IL-6, TNFα, CCL2, CCL3 and CXCL10 that further worsen inflammation. One possible explanation is that inflammatory proteins, including IL-6, are part of both an inflammatory loop and host responses to the infection, similar to other conditions like endocarditis and sepsis [1, 181]. The level of cytokine storm, as well as lymphopenia, are considered biomarkers of COVID-19, and are commonly used to predict the subsequent disease severity and mortality [182]. As a result, guidelines for the diagnosis and treatment of COVID-19 (first published in January 2020) recommended an early cytokine levels monitoring to reduce mortality [183]. One of the first studies to evaluate the serum cytokine storm in COVID-19 patients showed that several pro-inflammatory mediator levels, such as TNFα, IL-2, IL-7, IL-10, G-CSF, CXCL10, CCL2 and CCL3, were increased in ICU (*n* = 13) compared to non-ICU COVID-19 patients (*n* = 28) [184]. This was confirmed in a bigger sample by Diao et al. who evaluated COVID-19 patients (*n* = 522) and found increased levels of IL-6, IL-10, and TNFα compared to healthy subjects (*n* = 40) [91]. They positively correlated with severity and mortality in COVID-19 patients admitted to ICU (*n* = 43) compared to non-ICU admitted patients (*n* = 479). Similarly, another study confirmed that several serum pro-inflammatory mediators including IL-2R, IL-6, IL-10, and TNFα are increased in severe (*n* = 11) compared to moderate (*n* = 10) COVID-19 patients, while serum IFNγ levels were decreased negatively correlating with disease severity [92]. Increased serum IL-6 levels were found in non-surviving (*n* = 54) compared to surviving (*n* = 137) COVID-19 patients [185]. Similarly, serum IL-6, CXCL8, TNFα levels were increased in hospitalized COVID-19 patients (*n* = 1484) compared to non-infected by SARS-CoV-2 (*n* = 257) and serum levels of these pro-inflammatory mediators were positively correlated with mortality [186]. SARS-CoV-2 infection induced the cytokine storm development driving the pathogenesis of several severe manifestations of COVID-19 such as c-ARDS, thromboembolic diseases, encephalitis, acute kidney injury and vasculitis [93]. Nevertheless, another study showed that in paediatric/young COVID-19 patients (*n* = 65, < 24 years) who generally suffer less severe disease there were increased serum levels of IFNγ and IL-17A but not of IL-6 and TNFα protein compared to adult COVID-19 patients (*n* = 65). In addition, serum IFNγ levels were negatively correlated to disease severity [187].

Another study has confirmed these data showing that serum levels of several pro-inflammatory mediators such as IL-6, CXCL8 and TNFα and also serum NETs levels are increased in COVID-19 patients (*n *= 27), compared to healthy subjects (*n* = 12) [86].

IFNγ serum levels in SARS-CoV-2 infected patients are also closely related to the development of clinical symptoms, causing flu-like symptoms as general malaise, headaches, fever and fatigue, but increased serum INFγ levels is usually correlated to non-severe COVID-19 [188]. Serum CCL2, CCL3, CCL7, CXCL9, CXCL10, IL-1β and IL-1Ra levels increased in critical COVID-19 patients (*n* = 11) when compared to both severe COVID-19 patients (*n* = 25) and moderate COVID-19 patients (*n *= 14). Moreover, serum levels of these pro-inflammatory mediators were positively correlated to risk development and severity of acute respiratory failure [189].

A recent study has also showed that aging can alter several cytokines expression during COVID-19; in particular, hospitalized COVID-19 with > 70 years (*n* = 23) were characterized by increased serum IL-6 levels and decreased serum IFNγ levels, compared hospitalized COVID-19 with < 60 years (*n* = 26). These features, as previously reported, are correlated to poor prognosis [95].

Finally, lung tissue, in particular the alveolar cells type 2, of COVID-19 patients expressed increased senescence markers including p16^INK^ that positively correlated with expression of pro-inflammatory mediators such as IL-1β and IL-6 compared with lung tissue of patients with pneumonia not correlated with SARS-CoV-2 infection; these data were also confirmed with an in vitro analysis using alveolar cells type 2 infected with SARS-CoV-2 [44]. Although the release of inflammatory mediators significantly contributes to respiratory dysfunction in COVID-19, the lungs are not the only organs affected. Indeed, the sustained systemic inflammatory responses can also cause multiple organ failure, particularly in high-risk individuals such as those affected by DM, obesity and systemic arterial hypertension [190]. Because the severity of COVID-19 is associated with altered cytokine release, immunomodulatory therapies are used and/or proposed to control these responses [1, 191, 192].

### Immunomodulation in COVID-19

The severity of COVID-19 may be largely due to a dysregulated inflammatory response. Progression of the host immune response, that follows the initial phase of viral replication, leads an increased release of pro-inflammatory cytokines which could exacerbate c-ARDS and multiorgan failure [193–195]. According to this evidence, several immunomodulator drugs are currently strongly recommended in the treatment of severe COVID-19 [195]. However, the use of several antiviral drugs (alone or in combination) in hospitalized COVID-19 patients such as remdesivir, hydroxychloroquine, lopinavir, IFNβ and polyclonal convalescent plasma, have not shown improvement in terms of mortality, need of mechanical ventilation and length of hospitalization when compared to standard care. However, there are new compounds/drugs in ongoing clinical trials that may have benefits in these patients [195–198].

### Systemic glucorticoids

Although glucocorticoids, such as dexamethasone, are not specific drugs, these can act as immunomodulators and growing evidence show that systemic glucocorticoids reduce mortality among patients with severe COVID-19. In some trials, these appeared to have synergistic effects with IL-6R antagonists [195] and JAK blockers.

The use of dexamethasone (6 mg once daily for 10 days) in addition to standard care reduced mortality in hospitalized COVID-19 patients (*n* = 2104), compared to hospitalized COVID-19 patients treated only with standard care (*n* = 4321). However, only COVID-19 patients treated with dexamethasone who required mechanical ventilation and/or oxygen therapy showed a significant decreasing of mortality rate [199].

Another study investigated methylprednisolone in hospitalized COVID-19 patients (*n* = 62) and found a decreased risk of c-ARDS development and need of mechanical ventilation, compared to COVID-19 patients that were not treated with methylprednisolone (*n* = 139) [200]. Moreover, the use of dexamethasone (20 mg intravenously daily for 5 days, followed by 10 mg intravenously daily for additional 5 days or until ICU discharge) in addition to the standard care in hospitalized COVID-19 patients (*n* = 151) decreased the need for mechanical ventilation compared to hospitalized COVID-19 patients treated with only the standard care (*n* = 148) [201].

Furthermore, another study has shown the key role of the timing of starting systemic glucocorticoids therapy, use of early dexamethasone 6 mg/die i.v. in hospitalized COVID-19 patients within 24 h of ICU admission (*n* = 372) was correlated to decreased risk of acute respiratory failure development and the need for mechanical ventilation, compared to hospitalized COVID-19 patients treated much later with dexamethasone 6 mg/die i.v. [202]. However, no further beneficial effect was found when using an increased dose of dexamethasone (12 mg/daily i.v.) in severe COVID-19 patients (*n* = 503) as compared to the standard dose (*n* = 497) (6 mg/daily i.v.) [203].

### Targeting IL-1β, IL-6 signalling pathways

**Fig. 4:**
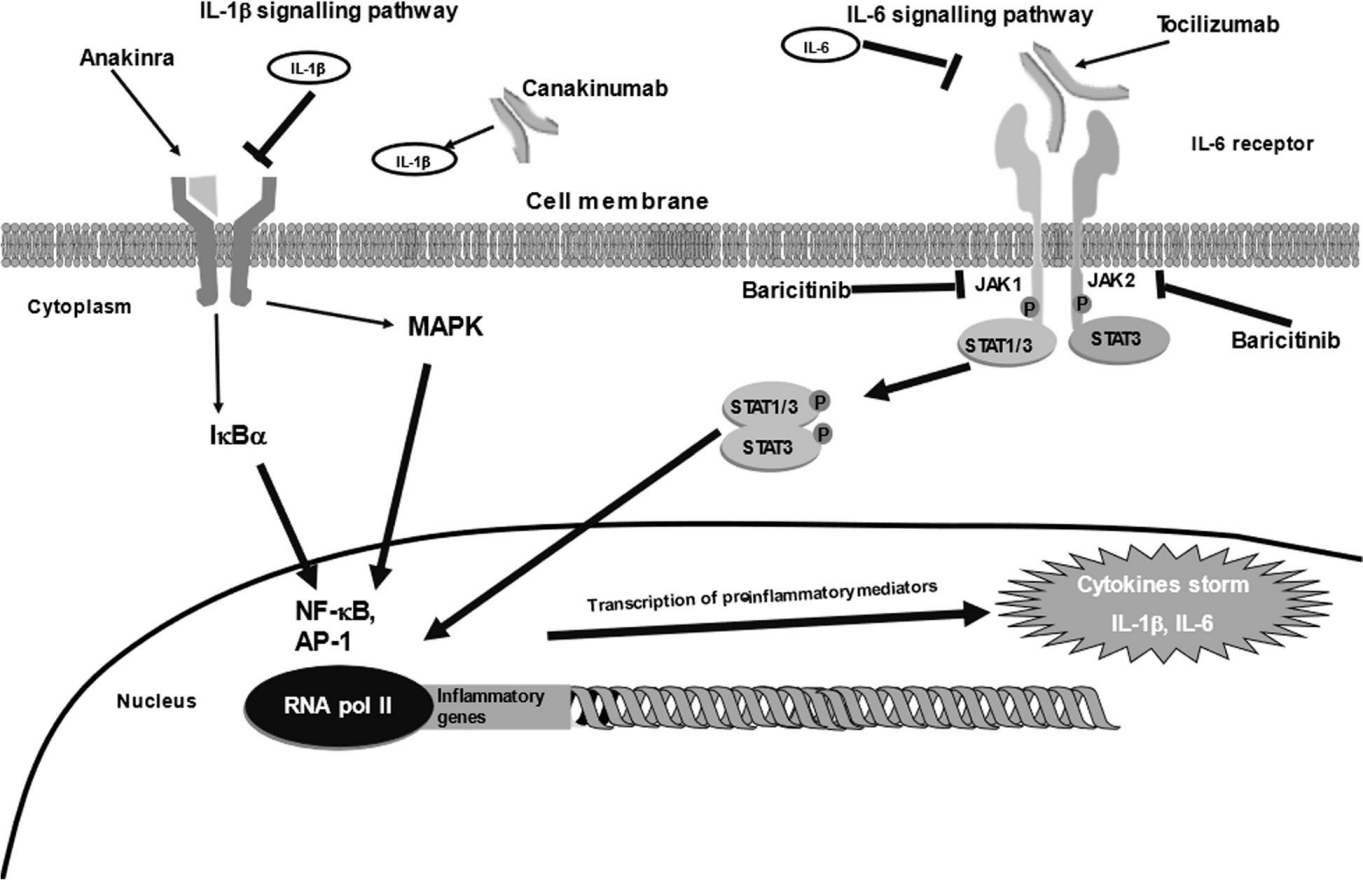
Immunomodulation in COVID-19 targeting IL-1β and IL-6 pro-inflammatory signalling pathways. Anakinra is a soluble IL-1Ra that acting as a decoy receptor binds IL-1β and prevents the activation of its receptor. Tocilizumab is a monoclonal antibody against IL-6R, blocking IL-6 binding to it and Canakinumab is a monoclonal antibody that bind IL-1β preventing activation of its receptor. Baricitinib is a dual JAK1 and -2 blocker. All these drugs inhibit the activation of several intracellular pro-inflammatory signalling pathways that induce the gene expression and the release of several pro-inflammatory mediators during SARS-CoV-2 infection that develops into a “cytokines storm”. Abbreviations: AP-1: activator protein-1; IκBα: inhibitor of kappa light chain gene enhancer in B cells; IL: interleukin; JAK: janus kinase; MAPK: mitogen-activated protein kinase; NF-κB: nuclear factor kappa B; P:phosphate; RNA: ribonucleic acid; STAT: signal transducer and activator of transcription

An increased and abnormal release of several pro-inflammatory mediators, including IL-1β and IL-6, has been found [195] in the dysregulated inflammatory responses of c-ARDS. Consequently, several drugs/compounds targeting IL-1β or IL-6 have been evaluated in several phase 3 clinical trials each (NCT04372186, NCT04409262, NCT04678739, NCT04364009, NCT04443881 and NCT04324021) as therapeutic option of SARS-CoV-2 infection to treat or prevent severe COVID-19 evaluated. Currently, both inhibitors of both IL-β1 and IL-6 pathways were approved for the treatment of hospitalized severe COVID-19 patients [204] (Fig. 4). Several clinical trials have evaluated also the use of anakinra, an anti-IL-1β drug. A study in severe COVID-19 patients (*n *= 59) using anakinra 200 mg twice for 3 days and after 100 mg twice for 1 day and 100 mg/die for 1 day, did not show any improvement in terms of survival, improvement of clinical symptoms and in decreasing need of mechanical ventilation compared standard care [205].

Conversely, a clinical study in hospitalized COVID-19 patients (*n* = 12, i.v. 300 mg/die for 5 days and then then tapered with lower dosing over 3 days) has shown a clinical improvement with a decreased need of oxygen therapy with or without mechanical ventilation, as compared to COVID-19 patients treated with standard care (*n* = 10) [206]. These finding were confirmed by Balkhair et al., which used anakinra (subcutaneous 100 mg twice daily for 3 days and then by 100 mg daily for 7 days) in hospitalized COVID-19 patients (*n* = 45) and found a decreased need of invasive mechanical ventilation, as compared to hospitalized COVID-19 patients (*n* = 24) treated with standard care [207]. Moreover, anakinra (subcutaneous 100mg once daily for 10 days) in hospitalized COVID-19 patients (*n* = 130) was also associated with a decreased risk of acute respiratory failure onset compared to hospitalized COVID-19 patients treated with standard care (*n* = 130) [208]. Finally, in hospitalized COVID-19 patients (*n* = 52), the administration of anakinra (subcutaneous 100 mg twice a day for 3 days, then 100 mg daily for 7 days) decreased risk of need of mechanical ventilation and mortality compared to hospitalized COVID-19 treated with standard care (*n* = 44) [209].

The use of another inhibitor of IL-1β in severe COVID-19 patients, such as canakinumab, (n = 227; intravenously injected 450 mg for body weight of < 60 kg, 600 mg for 60–80 kg, and 750 mg for > 80 kg) did not reduce mortality or the need of invasive mechanical ventilation compared to the placebo group (*n* = 227) [210].

Immune dysregulation may be influenced by IL-6, as suggested by the correlation between its levels and SARS-CoV-2 viremia and COVID-19 severity [211]. Consequently, research has focused on inhibitors to interrupt this inflammatory cascade. Tocilizumab, a monoclonal antibody against IL-6R used to treat multiple inflammatory diseases, improved outcomes in patients with severe COVID-19 [212–214]. The use of tocilizumab (i.v. 8 mg/Kg not to exceed 800 mg) in hospitalized COVID-19 patients that did not receive mechanical ventilation (*n* = 249), decreased the risk of need of mechanical ventilation during hospital-stay as compared to COVID-19 patients treated with placebo (*n* = 128) [215]. Similarly, tocilizumab (i.v. 8 mg/Kg) in hospitalized COVID-19 not requiring mechanical ventilation or ICU admission (*n* = 63), decreased the risk of need of mechanical ventilation as compared to hospitalized COVID-19 patients treated with standard care (*n* = 67), however, tocilizumab treatment did not decrease mortality [216].

Conversely, a large study evaluating tocilizumab in COVID-19 patients (*n* = 2022, 800 mg if weight > 90 kg; 600 mg if weight > 65 and ≤ 90 kg; 400 mg if weight > 40 and ≤ 65 kg; and 8 mg/kg if weight ≤ 40 kg) showed a decreased mortality, length of hospitalization and need of invasive mechanical ventilation compared to placebo group (*n* = 2094) [217]. Also, in critical ICU COVID-19 patients use of tocilizumab (*N* = 353, 8 mg/kg i.v. 1 or 2/die) decreased mortality compared to critical ICU COVID-19 patients treated with standard care (*n* = 402) [218]. In addition, a meta-analysis evaluating 27 trials has confirmed the effectiveness of tocilizumab, where use of this anti-IL-6 antibody decreased 28-day all-cause mortality in patients hospitalized for COVID-19 [219]. Conversely, a clinical trial has shown that the use of tocilizumab (i.v. 8 mg/Kg not to exceed 800 mg) in hospitalized COVID-19 patients (*n* = 161) did not induce clinical improvement and decrease risk of intubation or mortality, compared to hospitalized COVID-19 patients treated with placebo (*n* = 82) [220]. Similarly, in another cohort of hospitalized COVID-19 patients, use of tocilizumab (*n* = 294, 8 mg/Kg daily i.v.) did not improve clinical status and decrease hospital stay and mortality when compared to hospitalized COVID-19 patients treated with standard care (*n* = 144) [221].

Finally, another study has demonstrated that use of tocilizumab in COVID-19 patients (*n *= 79) was correlated with decreased need of mechanical ventilation and blood total leukocytes and neutrophils count and serum CRP levels even with several dosages such as < 400 mg/die, 400–800 mg/die and > 800 mg/die and with different timing of starting treatment such as 1–7 days post symptoms onset, 8–15 days, or more than 16 days, compared to COVID-19 treated with standard care. However, significantly decreased mortality was detected only in COVID-19 patients treated with 400–800 mg/die during the 8–15 days post symptoms onset [222].

Targeting other cytokine such as IL-37 and IL-38 represents a novel potential therapeutic option whereby their inhibition can decrease IL-1β release [93].

### Targeting kinases

Inhibitors of janus kinase (JAK) block the intracellular signalling pathways of cytokines that promote and sustain inflammation in severe COVID-19, including IL-2, IL-6, IL-10, GM-CSF and IFNγ [195]. Baricitinib is currently used in COVID-19 treatment, this is a selective JAK inhibitor (JAK1 and JAK2) that has shown to indirectly decrease IL-6 production [195, 223] (Fig. 4). Use of baricitinib (4 mg twice/die for 2 days and after 4 mg/die for 7 days) in hospitalized COVID-19 patients (*n* = 20), decreased serum levels of both IL-1β and IL-6 and moreover, increased the P/F ratio decreasing need of oxygen therapy as compared to hospitalized COVID-19 (*n* = 56) treated with standard care [224]. Similarly, baricitinib treatment (4 mg twice/die for 2 days and after 4 mg/die for 7 days) in addition to systemic glucocorticoids in hospitalized COVID-19 with acute respiratory failure (*n* = 62), was correlated with increased PaO2/FiO2 ratio at discharge time compared to hospitalized COVID-19 with acute respiratory failure (*n* = 50) treated only with systemic glucocorticoids [225]. A randomized trial evaluated the combination of baricitinib plus remdesivir (anti-viral) in > 1,000 hospitalized patients with COVID-19, finding that the combination of both drugs was superior to remdesivir alone in reducing recovery time and improving clinical status, particularly among non-intubated patients [226]. Moreover, a phase 3 clinical trial has shown that use of baricitinib (4 mg/day for 14 days) in hospitalized COVID-19 patients (*n* = 764) in addition to standard care decreased 28-day and 60-day mortality compared to hospitalized COVID-19 patients treated only with standard care [227]. Several ongoing, or just completed, clinical trials are further evaluating the efficacy of baricitinib (NCT04970719, NCT04693026, NCT05074420, NCT05082714).

Tofacitinib (10 mg twice for 14 days) has also shown some effect in hospitalized COVID-19 patients. It showed, in addition to standard care, a decrease in mortality and onset of respiratory failure (*n* = 114) when compared to hospitalized COVID-19 patients (*n* = 145) treated only with standard care [228].

### Prevention of thrombosis in COVID-19 patients

As previously described, COVID-19 is characterized by a pro-thrombotic status induced by both a SARS-CoV-2 induced endothelial cell damage and an inhibition of fibrinolysis [152] correlating to increased risk of coagulopathy and venous thromboembolism development. For these reasons, use of anticoagulant drugs and in particular low-molecular-weight heparin, was evaluated to improve outcomes of COVID-19 patients [229].

Although low-molecular-weight heparin is an anticoagulant drug, it is also known that can indirectly induce anti-inflammatory effects through binding and modulating pro-inflammatory mediators including; CXCL8, platelet growth factor 4 (PGF4) and the pro-inflammatory transcription factor nuclear factor kappa-B (NF-κB) both in endothelial and bronchiolar cells. This could explain how use of this therapy in COVID-19 patients improves their outcomes [229].

Currently, use of low-molecular-weight heparin at a thromboprophylaxis dosage is recommended in all hospitalized patients with pneumonia induced by SARS-CoV-2 infection.

However, a clinical trial has shown that the use of low-molecular-weight heparin at therapeutic dosages in critically ill COVID-19 patients (*n* = 536) did not improve survival, nor did it decrease the risk for ventilation support when compared to low-molecular-weight heparin at a thromboprophylaxis dosage (*n* = 567) [230].

Conversely, in non-critically ill COVID-19 patients (*n* = 1181) use of low-molecular-weight heparin at therapeutic dosages was correlated to decreased mortality and the need for mechanical ventilation, compared to non-critically ill COVID-19 patients (*n* = 1050) treated with low-molecular-weight heparin at a thromboprophylaxis dose [231]. Finally, use of enoxaparin in hospitalized COVID-19 patients (*n* = 96), was correlated to decreased mortality compared to hospitalized COVID-19 patients (*n* = 96) treated with unfractionated heparin [232].

A further clinical trial has shown that use of low-molecular-weight heparin at thromboprophylaxis dosages did not induce any benefits in non-hospitalized SARS-CoV-2 infected patients (*n* = 105), compared to standard treatments (*n* = 114) [233].

Similarly, use of low-molecular-weight heparin at thromboprophylaxis dosages in non-hospitalized patients did not decrease need of hospitalization in SARS-CoV-2 infected patients (*n* = 234) compared to SARS-CoV-2 infected patients (*n* = 238) treated with standard care [234].

## Conclusion

Although the COVID-19 pandemic still represents a major challenge for the healthcare systems worldwide, the cellular and molecular pathogenic pathways correlated to severe SARS-CoV-2 infection are still incompletely understood. There is growing evidence that c-ARDS is characterized by different cellular and molecular inflammatory pathways, compared to “classical” ARDS. This knowledge has therapeutic relevance for understanding the molecular basis of current drug treatments for c-ARDS and the search for novel compounds. For this reason, further investigations are needed to fully clarify the pathogenesis of this disease and to increase the clinical efficacy of our treatments.

## Data Availability

Not applicable.

## Abbreviations

ACE: Angiotensin I-converting enzyme
ADAMTS: Thrombo spondin type 1 motif member
ALI: Acute lung injury
Ang2: Angiopoietin-2
ARDS: Acute respiratory distress syndrome
BAL: Bronchoalveolar lavage
BMI: Body mass index
c-ARDS: COVID-19-related ARDS
CC16: Club cell secretory protein 16
CCR: Chemokine CC-motif receptor
CD: Cluster differentiation
COPD: Chronic obstructive pulmonary disease
COVID-19: Coronavirus disease 19
CS: Cigarette smoke
CXCL: Chemokine ligand
DAD: Diffuse alveolar damage
DM: Diabetes mellitus
FABP4: Fatty acid-binding protein 4
FUT2: Fucosyltransferase 2
GM-CSF: Granulocyte–macrophage colony-stimulating factor
HLA: Human leukocyte antigen
IPF: Idiopathic pulmonary fibrosis
IFI: Interferon inducing protein
IFITINIB1: Interferon-induced protein with tetratricopeptide repeats
IFITM: Interferon-induced transmembrane protein
IFN: Interferon
IFNA10: Interferon-alpha-10
IFNAR2: Interferon -alpha, -beta and -omega receptor 2
Ig: Immunoglobulin
IL: Interleukin
IL-10R: IL-10-receptor-beta
IRF7: IFN regulatory factor 7
JAK: Janus kinase
KL-6: Krebs von den Lungen-6
NF-κB: Nuclear factor kappa-B
NK: Natural killer
OAS: Oligoadenylate synthetases
PAI: Plasminogen activator inhibitor
PGF4: Platelet growth factor 4
PMA: Phorbol 12-myristate 13-acetate
PT: Prothrombin time
ROS: Reactive oxygen species
S100: Calcium binding protein
SARS-CoV-2: Severe acute respiratory syndrome-coronavirus-2
SNPs: Single nucleotide polymorphisms
SP: Surfactant proteins
SPP1: Secreted phosphoprotein 1
sRAGE: Soluble receptor for advanced glycation end products
STAT: Signal transducer and activator of transcription
TF: Tissue factor
TLR: Toll-like receptor
TMPRSS2: Transmembrane serine protease 2
TNF: Tumor necrosis factor
TREM1: Triggering receptor expressed on myeloid cells
VCAM: Vascular cell adhesion molecule
VEGF: Vascular endothelial growth factor
vWF: Von Willebrand factor
